# Dominant omodysplasia—A sporadic case—A new case report and review of the literature

**DOI:** 10.1002/ccr3.6187

**Published:** 2022-08-03

**Authors:** Aidin Arabzadeh, Behnam Baghianimoghadam, Mohammad Hossein Nabian, Yousef Fallah, Mohammad Mehdi Ebrahimnasab

**Affiliations:** ^1^ Department of Orthopedic Surgery Tehran University of Medical Sciences, Imam Khomeini Hospital Complex Tehran Iran; ^2^ Department of Orthopedic Surgery, Shariati Hospital Tehran University of Medical Sciences Tehran Iran; ^3^ Department of Orthopedic Surgery, Sina Hospital Tehran University of Medical Sciences Tehran Iran

**Keywords:** criteria, diagnosis, dominant omodysplasia

## Abstract

Omodysplasia is an extremely rare skeletal dysplasia. Since introducing this phenotype as a new syndrome, ten cases of the autosomal dominant type of this disease have been reported. Here, we present a new patient and review published articles in this field to provide a clinical diagnostic criterion.

## 1 INTRODUCTION

Omodysplasia is a rare skeletal dysplasia characterized by short limbs (especially short upper limbs) and craniofacial dysmorphism (including hypertelorism and a depressed nasal bridge with a short nose and a long philtrum). Both autosomal recessive (OMOD1; OMIM 258315) and dominant types (OMOD2; OMIM 164745) are described. Therefore, genetic transmission and genetic heterogeneity are important.^1^, ^2^, ^3^, ^4^

Homozygous or heterozygous compound mutations have been observed in the heparan‐sulfate proteoglycan glypican six gene (GPC6) in the recessive type 3. De novo Heterozygous nonsense mutations (p.Trp548 *) in the FZD2 gene have also recently been described for the dominant type.^5^, ^6^, ^7^, ^8^

Since introducing this phenotype as a new syndrome, 10 cases of the autosomal dominant type of this disease have been reported. In this case report, we present a new patient. We also review published articles in this field to provide a clinical diagnostic criterion.

### 1.1 Case presentation

The patient was a 6‐year‐old boy from a non‐consanguineous marriage who had been referred to a hand surgery clinic due to a short both upper limbs. The patient was an Afghan refugee living in Iran. Complete information on the patient's condition at birth was not available. Based on a history taken from the patient's mother, there was no history of such a problem in any patient's family members. The patient's mother and father were healthy.

He has a history of cleft lip surgery in infancy. He also had gastroesophageal reflux disease and underwent surgery. On examination, short arms were overt. Shoulder and hand movements were regular. There was an impaired left elbow extension (about 30 degrees flexion contracture), but no problem in the right elbow was seen. Both sides' forearm supination and pronation movements were normal (87 degrees supination and pronation for both sides).

In the genital examination, the patient had micropenis and cryptorchidism, which needed surgery, and was referred to a pediatric surgeon.

The patient had 147 cm height (about 50 percentile based on WHO height for age chart^9^), and his intelligence was normal.

Also, dysmorphic facial features were seen, including a prominent forehead, round face, hypertelorism, a depressed nasal bridge with a short nose and a long philtrum, bilateral cleft lip, and anteverted nares.

**FIGURE 1:**
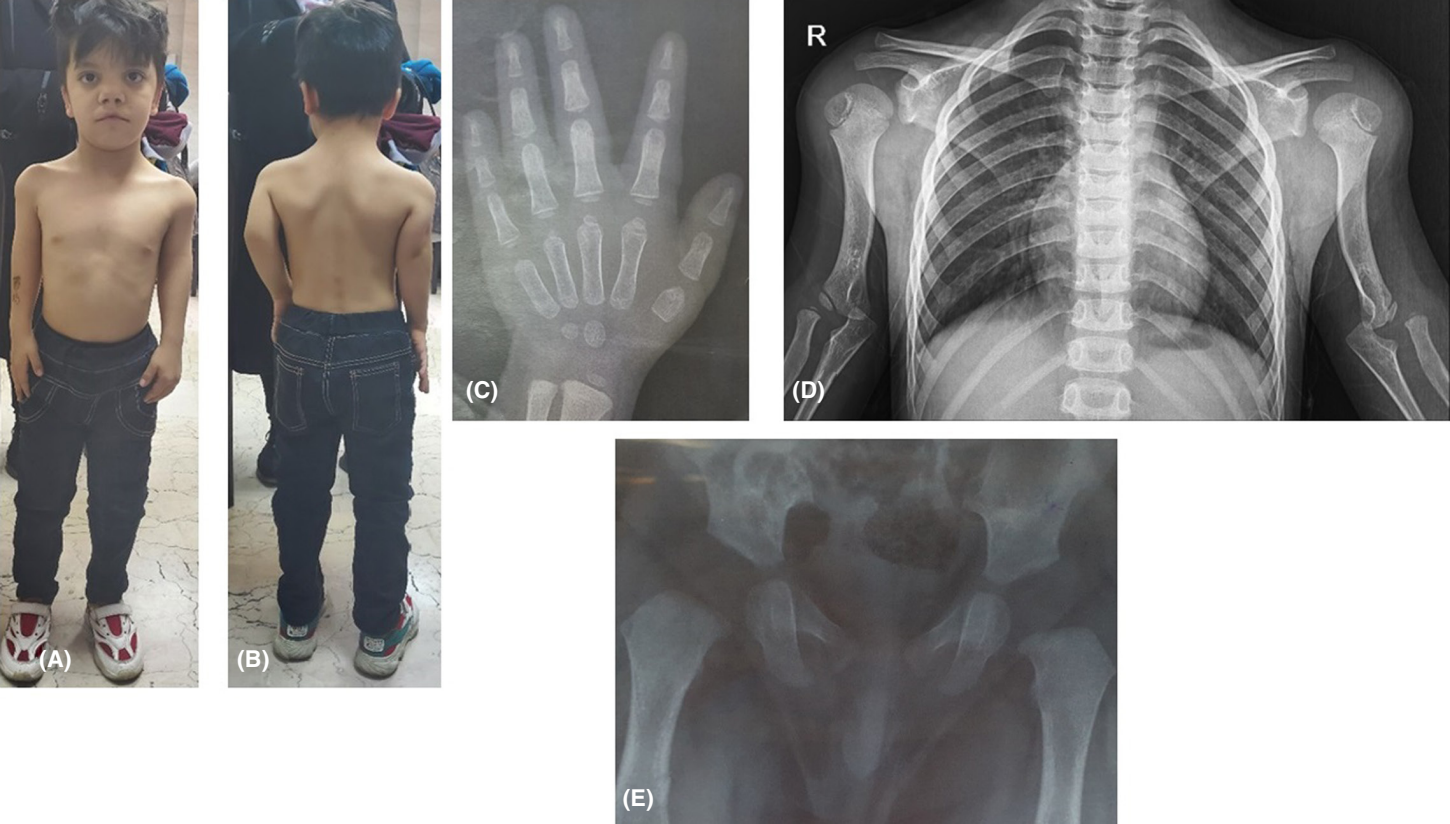
Characteristics of patient. Short humerus, dysmorphic facial features including a large forehead, round face, hypertelorism, depressed nasal bridge with short nose and a long philtrum, cleft lip, and anteverted Nares. The height of patient was normal (A), limited extension in left elbow (B), short first metacarpal on right hand radiography (C), Bilateral radial head dislocation and proximal radioulnar joint disatasis in chest X‐ray (D). Normal hip radiography (E). The C and E radiographies are for when the patient was 4‐year‐old

In the radiographic study, both humeruses were short. The condyles, especially the left condyle, were hypoplastic and had a distally tapered view. There was a bilateral dislocation of the upper radioulnar joint. In radiography, the first metacarpal was short bilaterally. The lower limb radiograph was normal (Figure 1).

## 2 DISCUSSION

In 1989, Maroteaux and colleagues^1^ first described omodysplasia syndrome by introducing five patients. The common features of all these patients were short humerus, dislocation and diastasis of the upper radioulnar joint, and similar abnormal craniofacial symptoms.

Two patients in this study were previously diagnosed with Robinow syndrome.^10^ The first three patients in the Maroteaux study were not of short stature. According to the authors, a mother and child among them reinforced the suspicion of autosomal dominant inheritance. Despite being similar in their upper limbs to the first three, the following two patients also had short lower limbs and were micromelic dwarfs. They appeared to be autosomal recessively involved.

All patients had some degree of genitalia disorder, and the authors even explained differences in the upper limb phenotype of the first three patients with the next two. For example, the first metacarpal was short in the first three patients, while the following two patients had a normal first metacarpals.^1^

Venditti and colleagues^2^ identified a mother and son who had symptoms similar to the first three patients in the Maroteaux study. Venditti confirmed that in boys with dominant omodysplasia, genital hypoplasia is a significant symptom. This symptom was also seen in Maroteaux patients. Hypoplasia of the labia majora and clitoris was also seen in the female patient of the Venditti study. The development of motor and intelligence in the mother was completely normal, and there was no previous family history.

Many patients with omodysplasia were previously diagnosed with Robinow syndrome.^8^ Robinow syndrome was first described as an autosomal dominant disease.^11^ Later, the autosomal recessive type was introduced.^12^ There are many similar phenotypic symptoms between the two syndromes. The main symptoms of Robinow syndrome include the followings: mesomelic brachymelia, hypoplastic genitalia, craniofacial anomalies (i.e., hypertelorism, short‐upturned nose, anteverted nose, frontal bossing, and broad nasal bridge). Short stature is ordinary. Radiographs show spinal abnormalities, rib abnormalities, radial head dislocation, brachydactylia, and bifid terminal phalanges.^13^ In patients with dominant omodysplasia, height is often standard, the elbow is always abnormal, the first metacarpal is short, and patients have isolated upper extremity rhizomelic brachymelia.

The autosomal recessive type of omodysplasia is associated with severe short stature and developmental delay. At the same time, the height is normal or close to normal in the dominant type, and the patient's intelligence is also normal. The normal first metacarpal length and the presence of bulky proximal end and distal hypoplasia of the femur in radiography (club‐like appearance) are in favor of the autosomal recessive type of omodysplasia.^14^

Saal et al.^5^ introduced a new mother and daughter with dominant omodysplasia. By genetically examining and whole‐exome sequencing and comparing them with non‐involved family members, they found a new mutation in an FZD2 in the affected individuals.

This gene mutation was confirmed in three other studies by Türkmen S,^6^ Warren HE,^7^ and Nagasaki K.^8^ Türkmen,^6^ as well as Saal,^7^ had bilateral cleft lip or palate in their patients. This problem was not mentioned in previously reported patients. Our patient also had a history of cleft lip surgery.

The presence of the short first metacarpal is a typical finding in autosomal dominant omodysplasia. However, Türkmen and Venditti have also reported the presence of a shortened middle phalanx of the 5th finger in their patients, which was not mentioned in previous patients and our patient.

Genital hypoplasia is seen in almost all male patients and some female patients. Our patient also had a micropenis. The complete clinical features of all reported cases are presented in Table 1.

**TABLE 1:** Clinical feature of OMD2 patients

feature	Present patient	Nagasaki et al (2018)	Turkmen et al (2017)	Saal et al (2015)		Gordon et al (2014)	Venditti et al (2002)		Maroteaux et al. (1998)		
				Patient 1	Patient 2		Patient 1	Patient 2	Patient 1	Patient 2	Patient 3
Age of last examination	6 years	15 years	21 years	25 years	6 years	48 years	25 years	1 month	newborn	27 years	10 years
Sex	M	M	F	F	M	F	F	M	M	F	F
Height, Percentile	25	25	<5	10	<5	<5	25	25	5	10	10
Facial feature											
Round face	+	−	+	+	+	−	+	NA	+	NA	+
Prominent forehead	+	+	+	+	+	+	+	+	−	−	+
Hypertelorism	+	+	+	−	−	−	+	+	+	+	−
Broad nasal bridge	+	+	+	+	+	+	+	+	+	−	−
Small nose with broad nasal tip	+	+	−	+	+	+	+	+	+	+	+
Long philtrum	+	+	−	+	+	+	+	+	−	−	−
Microretrognathia	−	−	+	+	+	+	−	+	−	−	−
Cleft lip/palate	+	−	+	+	−	−	−	−	−	−	−
Skeletal features											
Short humeri	+	+	+	+	+	+	+	+	+	+	+
Radial dislocation	+	+	+	+	+	+	+	+	+	+	+
Limited elbow joints	+	NA	+	+	+	+	−	NA	+	+	+
Short ulnae		−	+	NA	+	−	+	+	+	+	+
Short first metacarpal bones	+	+	+	NA	+	+	+	+	+	+	+
Short fibulae	−	−	+	NA	+	−	−	−	−	−	−
Genital anomalies	+	+	−	+	−	+	+	+	+	−	−
*FZD2* mutation	NA	(c.1640C>A, p.S547*)	c.1301G>T (p.Gln434Val)	c.1644G>A (p.Trp548*)		NA	NA	NA	NA	NA	NA

*Note:* Facial features, radial head dislocation, short first metacarpal, short humeri, and genital anomalies in male patients are frequently reported.

Abbreviation: NA, not Assessed.

In his study, Türkmen^6^ finally presented a diagnostic triad for autosomal dominant omodysplasia:

- Short upper limb with radial head dislocation and limited elbow extension.
- The first short metacarpal.
- The typical shape of the face (includes a prominent forehead and a short nose with a depressed broad nasal bridge).

According to what has been reported, the following two items can be added to this triad: (4) normal or close to normal height and (5) normal intelligence.

This pentad can be considered the diagnostic criterion of autosomal dominant type omodysplasia.

Genital disorders such as micropenis in male patients can be added to this criterion. Patients may or may not have a cleft lip or palate.

Due to the rarity of this disease, the diagnosis is more clinical than the laboratory. Therefore, the presence of this diagnostic criterion should raise suspicion about the existence of this syndrome. However, according to the existing research, the presence of a mutation in FZD2 and genetic testing can confirm the diagnosis.

### 2.1 Learning points

- We suggest the diagnostic pentad of (1) short upper limb with radial head dislocation and limited elbow extension, (2) the short first metacarpal, (3) the typical shape of the face (prominent forehead, short nose, and depressed broad nasal bridge), (4) standard height, and (5) normal intelligence for diagnosis of the autosomal dominant type omodysplasia.

## AUTHOR CONTRIBUTIONS

Aidin Arabzadeh and Behnam Baghianimoghadam had contribution in conception of the work, wrote the manuscript and translated it. Mohammad Hossein Nabian, Mohammad Mehdi Ebrahimnasab, and Yousef Fallah revised and approved the manuscript scientifically for submission.

## FUNDING INFORMATION

None.

## CONFLICT OF INTEREST

Not declared.

## CONSENT

Written informed consent was obtained from the patients' parents to publish this report in accordance with the journal's patient consent policy.

## Data Availability

The authors confirm that the data supporting the findings of this study are available within the article [and/or] its supplementary materials.
